# Spectral CT iodine quantification for peritoneal metastasis burden and resectability prediction in ovarian cancer: a retrospective cohort study

**DOI:** 10.3389/fonc.2026.1823515

**Published:** 2026-05-14

**Authors:** Yongfeng Liu, Xiaomin Wu, Huipeng Deng, Yihong Lin, Meiyan Lin

**Affiliations:** 1 Department of Gynecology, Longyan First Affiliated Hospital of Fujian Medical University, Longyan, Fujian, China; 2 Department of Radiology, Longyan First Affiliated Hospital of Fujian Medical University, Longyan, Fujian, China

**Keywords:** iodine quantification, ovarian cancer, peritoneal metastasis, spectral CT, surgical resectability

## Abstract

**Background and purpose:**

Accurate preoperative assessment of peritoneal metastasis burden in ovarian cancer remains challenging as conventional CT lacks sensitivity for small-volume disease. Spectral CT iodine quantification provides objective metrics of tumor vascularity that may predict surgical resectability. This retrospective study evaluated iodine-based parameters for estimating surgical Peritoneal Cancer Index (PCI) and predicting complete cytoreduction (R0).

**Materials and methods:**

We retrospectively identified 145 ovarian cancer patients who underwent preoperative triphasic spectral CT scans and subsequent cytoreductive surgery between June 2022 and December 2023. Two blinded radiologists quantified iodine concentration (IC), normalized iodine concentration (NIC), spectral curve slope (λHU), and effective atomic number (Zeff). Patients were stratified by surgical PCI into low (≤6), intermediate (7-15), and high (≥16) burden subgroups. Statistical analyses included correlation studies, ROC curves, and multivariable logistic regression.

**Results:**

Iodine-based parameters demonstrated a strong correlation with intraoperative PCI (IC: r=0.85, NIC: r=0.74, both P<0.001). NIC increased progressively across tumor burden categories (0.43, 0.57, 0.71; P<0.001). For predicting R0 resection, NIC achieved optimal performance (AUC = 0.88, 95% CI: 0.81-0.94). The cutoff value ≤0.55 yielded sensitivity 84.5%, specificity 79.5%, and a negative predictive value of 94.8% for identifying patients who would achieve R0 resection (i.e., among patients with NIC ≤0.55, 94.8% achieved R0). A combined model (NIC + PCI + CA-125) achieved AUC = 0.93 (95% CI: 0.87-0.97) with excellent interobserver reproducibility (ICC >0.84).

**Conclusion:**

Spectral CT iodine quantification demonstrates potential as a complementary tool for estimating peritoneal metastasis burden and predicting surgical resectability. The NIC threshold of 0.55 shows promise for stratifying patients, though external validation is needed before integration into preoperative staging protocols.

## 1 Introduction

Ovarian cancer represents the most lethal gynecologic malignancy, with approximately 70% of patients presenting at advanced stages characterized by widespread peritoneal metastasis (1, 2). This extensive peritoneal dissemination not only dictates the feasibility of surgical resection but also serves as the principal prognostic determinant, directly influencing the likelihood of achieving optimal outcomes (3). Consequently, the burden of peritoneal disease has become the cornerstone of treatment stratification. Current National Comprehensive Cancer Network guidelines recommend primary surgery for patients with resectable disease, aiming for complete macroscopic resection—a surgical endpoint that confers a significant and independent survival advantage (4, 5). However, accurate preoperative assessment of peritoneal tumor burden remains a formidable challenge, with conventional CT demonstrating inadequate sensitivity for small-volume disease (false-negative rates approaching 40% for lesions <5 mm) (6, 7). Consequently, 30% of patients undergo exploratory laparotomy only to be deemed unresectable intraoperatively, exposing them to unnecessary morbidity while delaying systemic therapy (8).

Spectral CT addresses this limitation through dual-energy acquisition and material decomposition algorithms, enabling quantitative assessment of tissue iodine concentration—a surrogate marker of tumor angiogenesis (9, 10). Unlike conventional CT’s qualitative interpretation, this technique provides objective metrics including iodine concentration (IC), normalized iodine concentration (NIC), spectral curve slope (λHU), and effective atomic number (Zeff), which have demonstrated diagnostic value in hepatocellular carcinoma, gastric cancer, and lung cancer (11, 12). The biological rationale is compelling: progressive tumor burden drives neovascularization, resulting in quantifiable iodine uptake elevation. Nevertheless, the clinical utility of spectral CT iodine quantification in ovarian cancer remains largely unexplored (13, 14).

To address this gap, we conducted a retrospective cohort study evaluating whether spectral CT-derived iodine parameters could serve as quantitative biomarkers for peritoneal metastasis burden. We hypothesized that iodine quantification would correlate strongly with surgical Peritoneal Cancer Index (PCI) and that specific thresholds could predict R0 resection. Secondary objectives included determining optimal cutoffs, developing a multivariable predictive model, assessing interobserver reproducibility, and constructing a clinically applicable decision algorithm.

## 2 Methods

### 2.1 Study design and ethics

This retrospective, single-center cohort study was conducted at the Gynecologic Oncology Center of our hospital between January 2022 and December 2024, with rigorous adherence to established clinical research standards. The protocol received approval from the Institutional Review Board (IRB No. LYREL2026-K011-01) to ensure transparency and protocol adherence. An independent Data Monitoring Committee (DMC) was established for the purpose of overseeing data quality assurance and ensuring compliance with the study protocol, given the inclusion of a prospectively defined imaging-surgical correlation component with predefined quality metrics. The DMC’s role focused exclusively on monitoring data integrity, image quality standards, and adherence to the ≤10-day CT-surgery interval. No interim efficacy analyses were performed, as this was not an interventional trial. All participants provided written informed consent for the surgical procedure and standard preoperative imaging. For the retrospective analysis of imaging data, the Institutional Review Board approved a waiver of additional written informed consent given the retrospective nature of the study and the use of de-identified data. The investigation strictly followed the Declaration of Helsinki principles, with particular attention to minimizing radiation exposure through optimized scan protocols and maintaining patient confidentiality via de-identified data management.

### 2.2 Study population and recruitment

Consecutive patients presenting with newly diagnosed, treatment-naïve ovarian cancer were screened for eligibility through a standardized enrollment process. Eligible participants were women aged 18–75 years with histopathologically confirmed primary epithelial ovarian, fallopian tube, or primary peritoneal carcinoma scheduled for primary cytoreductive surgery. All patients underwent systematic preoperative assessment including ECOG performance status evaluation, serum tumor marker profiling (CA-125, HE4, CA19-9), and comprehensive laboratory screening. A critical enrollment criterion mandated that the interval between preoperative spectral CT acquisition and surgical intervention not exceed 10 days, thereby minimizing disease progression bias and ensuring temporal validity of imaging-surgical correlations. Patients who received neoadjuvant chemotherapy were excluded from the primary analysis cohort but included in a separate sensitivity analysis to assess the generalizability of findings to this population. For patients whose surgical plan was altered to interval debulking based on intraoperative findings but who had not received preoperative neoadjuvant chemotherapy, they remained in the cohort for primary analyses based on initial treatment assignment (i.e., the principle of analyzing patients according to their planned primary treatment strategy). Exclusion criteria comprised: (i) prior abdominal or pelvic surgery that precluded accurate PCI assessment; (ii) history of other malignancies within 5 years; (iii) severe renal insufficiency (estimated glomerular filtration rate <30 mL/min/1.73m²) contraindicating iodinated contrast administration; (iv) known allergy to iodinated contrast agents; (v) pregnancy or lactation; (vi) inadequate spectral CT image quality due to motion artifacts, beam hardening, or incomplete anatomical coverage; (vii) interval >10 days between CT examination and surgery; and (viii) unavailable surgical or pathological data. Patients with neoadjuvant chemotherapy were excluded from primary analyses but included in a separate sensitivity analysis.

### 2.3 Spectral CT acquisition protocol

Imaging examinations were performed on a dual-source dual-energy CT scanner (SOMATOM Drive, Siemens Healthineers) after daily water phantom calibration. Scan range extended from the diaphragmatic dome to the pelvic floor. Dual-energy scanning used 80/Sn140 kVp with automated tube current modulation, 0.8 pitch, and 0.6 mm reconstruction thickness. A non-ionic iodinated contrast agent (ioversol 350 mgI/mL, 1.2 mL/kg at 3.5 mL/s) was administered intravenously, followed by a 30 mL saline flush. Triphasic acquisition included arterial (30–35 s), venous (60–70 s), and delayed (120–180 s) phases. The venous phase was used as the primary dataset for analysis given its optimal tumor conspicuity.

### 2.4 Image post-processing and iodine quantification

Raw projection data were transmitted to a dedicated spectral imaging workstation (syngo.via VB60) for material decomposition and generation of iodine density maps, virtual monoenergetic images (40–140 keV), and effective atomic number maps. Quantitative analysis was performed by two board-certified abdominal radiologists (10 and 9 years pf experience) who underwent standardized training sessions using a pilot cohort of 30 cases to harmonize measurement techniques. A semi-automated ROI placement protocol was implemented to enhance reproducibility: contiguous voxels exceeding a threshold of 1.0 mg/mL iodine concentration within segmented lesions were automatically identified, subsequently refined by manual exclusion of necrotic or cystic components based on morphological criteria and HU values (<20 HU on non-contrast images). Each ROI encompassed at least 10 mm² of viable tumor tissue, with measurements performed in triplicate across different lesion sections and averaged. For multifocal peritoneal disease, quantitative parameters were derived from the three largest deposits in distinct PCI regions, with additional calculation of total peritoneal iodine burden (summation of all lesion IC × volume measurements) to capture global disease load. The abdominal aorta served as the reference standard for normalization, with ROIs positioned centrally within the vessel lumen at the level of the celiac trunk, avoiding atherosclerotic plaques. The primary spectral parameters included iodine concentration (IC, mg/mL), normalized iodine concentration (NIC), spectral curve slope (λHU), and effective atomic number (Zeff). Interobserver agreement was quantified using intraclass correlation coefficients (ICC), with ICC >0.80 required for inclusion in final analyses; discrepant cases were adjudicated by a third senior radiologist.

### 2.5 CT-based peritoneal cancer index assessment

To evaluate the predictive accuracy of spectral CT, two radiologists independently assigned an estimated CT-PCI score based on preoperative images using the standard Sugarbaker regional classification system. We acknowledge that detecting lesions <5 mm and reliably distinguishing between jejunum and ileum (upper vs. lower small bowel) on CT remains challenging. Spectral CT’s iodine quantification does not directly resolve these anatomical limitations; rather, it provides complementary functional information about lesion vascularity. For PCI region assignment, radiologists used anatomical landmarks (ligament of Treitz for jejunum, ileocecal valve for ileum) supplemented by coronal and sagittal reformats to improve localization accuracy. Each of the 13 abdominopelvic regions was scored according to lesion size on a 0–3 scale, with final CT-PCI calculated as the summation of regional scores. This preoperative, blinded assessment enabled direct comparison with intraoperative PCI measurements, facilitating calculation of diagnostic accuracy metrics including sensitivity, specificity, and predictive values for detecting high-burden disease (PCI ≥16). The correlation coefficient between CT-PCI and surgical PCI served as a key indicator of the clinical validity of spectral CT assessment.

### 2.6 Intraoperative surgical evaluation

All cytoreductive procedures were performed by a dedicated gynecologic oncology surgical team with standardized operative protocols and extensive experience in peritoneal surface malignancies. Systematic exploration of 13 abdominopelvic regions was performed in a predetermined sequence to ensure comprehensive assessment. Intraoperative PCI scoring was conducted by the primary surgeon and independently verified by a second attending surgeon, with real-time video documentation of all regions for subsequent quality assurance review. Cytoreductive outcomes were classified according to residual disease status: complete macroscopic resection (R0) defined as no visible tumor, R1 as residual implants <2.5 mm, and R2 as gross residual disease >2.5 mm. Comprehensive operative documentation encompassed procedural duration, estimated blood loss, transfusion requirements, extent of multivisceral resection, and postoperative complications graded by the Clavien-Dindo classification.

### 2.7 Pathological confirmation and biomarker analysis

Resected peritoneal specimens underwent comprehensive histopathological examination by subspecialty-trained gynecologic pathologists blinded to imaging interpretations. All lesions were confirmed as metastatic carcinoma through hematoxylin and eosin staining. Immunohistochemical profiling included a standardized panel of CA-125, CK7, WT-1, and PAX8 to validate ovarian primary origin, with additional markers (CK20, ER, PR) employed for differential diagnosis in selected cases. Tumor histotype, grade, and the percentage of viable malignant tissue within each specimen were systematically recorded, with results integrated into multivariable models to account for tumor biological heterogeneity.

A multi-level quality assurance framework was implemented. Technical quality control comprised daily water phantom spectral calibration, weekly CT number accuracy assessments, and semi-annual independent audits of radiation dose compliance. Radiologist performance was monitored through quarterly measurement reproducibility tests using a reference case library. PCI assessment quality was ensured through a two-tiered approach. First, all PCI assessments were reviewed monthly by a multidisciplinary tumor board to maintain consistency and validate scoring accuracy against video recordings. Second, an additional video-based peer review was performed on a random 20% of cases by an independent surgical reviewer not involved in the original assessment, with ongoing feedback to maintain PCI scoring reliability. These represent complementary rather than overlapping processes. All data were captured through a secure electronic case report form with built-in range checks and logical validations. Independent data monitoring was performed by a dedicated clinical trials unit, with source document verification for 100% of primary endpoint data and 20% of secondary parameters. An Independent Data and Safety Monitoring Board was convened to oversee data quality assurance, image quality standards, and adherence to protocol specifications, given the inclusion of a prospective imaging-surgical correlation component. The DSMB’s role focused on monitoring data integrity rather than traditional safety monitoring for an interventional trial; thus, no interim efficacy analyses were performed, and semi-annual reviews addressed data quality metrics only.

### 2.8 Statistical analysis

As this is a retrospective study, no formal sample size calculation was performed prior to data collection. Instead, we included all eligible patients meeting the predefined inclusion criteria during the study period. The achieved sample size (n=145, with 102 in the metastasis cohort) provided adequate statistical power for the planned analyses, as *post-hoc* calculation indicated that this sample size could detect a medium effect size (Cohen’s d=0.5) with >80% power at α=0.05 for discriminating R0 versus R1/R2 resection.

Statistical analyses were conducted using SPSS version 26.0 and R version 4.2.0. The primary analytical cohort was randomly divided into training (70%, n=102) and validation (30%, n=43) sets to facilitate model development and internal validation. Continuous variables were expressed as mean ± standard deviation or median (interquartile range) based on normality assessment using the Kolmogorov-Smirnov test, with group comparisons performed via independent t-tests or Mann-Whitney U tests. Categorical variables were presented as frequencies (percentages) and analyzed using χ² or Fisher’s exact tests.

Pearson or Spearman correlation coefficients were calculated between each iodine parameter (IC, NIC, λHU, Zeff) and surgical PCI scores to assess linear relationships. For each iodine parameter, ROC curves were constructed on the training cohort to evaluate diagnostic performance, with optimal cutoffs determined by maximizing the Youden index (Sensitivity + Specificity - 1). Sensitivity, specificity, positive predictive value (PPV), negative predictive value (NPV), and accuracy were calculated with 95% confidence intervals, where AUC confidence intervals employed the DeLong method and between-curve comparisons used DeLong’s test. Univariate logistic regression initially screened all iodine parameters, clinical variables (age, BMI, CA-125, HE4, histology, grade, FIGO stage), and radiological features (CT-PCI, omental cake presence), with variables achieving P<0.10 entered into multivariable analysis. Multivariable logistic regression was performed using backward elimination with P<0.05 retention criterion. The final combined model’s performance was assessed using AUC, calibration (Hosmer-Lemeshow test), and clinical utility through Decision Curve Analysis (DCA), with bootstrap resampling (n=1000) used for internal validation of model stability. The combined model developed on the training cohort was frozen and prospectively applied to the validation cohort without any refitting to assess generalizability, and patients were dichotomized using the NIC cutoff (≤0.55 vs. >0.55) identified from the training cohort to compare surgical outcomes (R0 rates) between groups using χ² test, with a decision algorithm constructed based on NIC threshold performance and its clinical impact simulated using validation cohort data.

## 3 Results

### 3.1 Patient characteristics and enrollment

Between June 2022 and December 2023, a total of 182 patients with suspected advanced ovarian cancer were screened for eligibility, of which 153 patients met inclusion criteria and provided informed consent. Of these, 8 patients were subsequently excluded due to inadequate image quality (n=3), interval >10 days between CT and surgery (n=4), or unavailable surgical data (n=1), yielding a final cohort of 145 patients for primary analysis. The mean interval from CT examination to surgery was 5.2 ± 2.1 days. Baseline demographic and clinical characteristics are summarized in **Table 1**. The median patient age was 58 years (interquartile range [IQR], 52–65 years), and 87.6% (127/145) had epithelial serous carcinoma. The median preoperative CA-125 level was 847 U/mL (IQR, 423–1,562 U/mL).

**Table 1:** Baseline demographic and clinical characteristics of the study cohort.

Characteristic	Total cohort (n=145)	Metastasis group (n=102)	Non-metastasis group (n=43)	P-value
Age (years)	58 (52–65)	59 (53–66)	56 (50–63)	0.18
BMI (kg/m²)	24.3 (21.8–27.1)	24.5 (22.0–27.3)	23.9 (21.5–26.8)	0.42
Histological type				0.76
Serous carcinoma	127 (87.6%)	90 (88.2%)	37 (86.0%)	
Endometrioid carcinoma	9 (6.2%)	6 (5.9%)	3 (7.0%)	
Clear cell carcinoma	5 (3.4%)	4 (3.9%)	1 (2.3%)	
Other	4 (2.8%)	2 (2.0%)	2 (4.7%)	
Tumor grade				0.65
Grade 1	8 (5.5%)	5 (4.9%)	3 (7.0%)	
Grade 2	31 (21.4%)	23 (22.5%)	8 (18.6%)	
Grade 3	106 (73.1%)	74 (72.5%)	32 (74.4%)	
FIGO stage (preoperative)				<0.001
I–II	8 (5.5%)	0 (0%)	8 (18.6%)	
III	118 (81.4%)	85 (83.3%)	33 (76.7%)	
IV	19 (13.1%)	17 (16.7%)	2 (4.7%)	
CA-125 (U/mL)	847 (423–1,562)	892 (456–1,645)	723 (385–1,287)	0.08
HE4 (pmol/L)	462 (298–785)	489 (312–823)	401 (275–698)	0.12
Surgical PCI	11 (6–17)	14 (9–19)	—	—
Cytoreductive outcome				—
R0 resection	58 (40.0%)	58 (56.9%)	—	
R1 resection	28 (19.3%)	28 (27.5%)	—	
R2 resection	16 (11.0%)	16 (15.7%)	—	

Data are presented as median (interquartile range) or number (percentage). BMI, body mass index; FIGO, International Federation of Gynecology and Obstetrics; PCI, Peritoneal Cancer Index.

Intraoperative assessment confirmed peritoneal metastasis in 102 patients (70.3%), constituting the metastasis group, while 43 patients (29.7%) had no macroscopic peritoneal disease. Within the metastasis cohort, the distribution according to surgical PCI was low-burden (PCI ≤6) in 31 patients (30.4%), intermediate-burden (PCI 7–15) in 41 patients (40.2%), and high-burden (PCI ≥16) in 30 patients (29.4%). Complete cytoreduction (R0) was achieved in 58 patients (56.9%) within the metastasis group, while 44 patients (43.1%) had residual disease (R1 in 28, R2 in 16). The median surgical PCI for the entire cohort was 11 (IQR, 6–17).

### 3.2 Iodine quantification in metastatic versus non-metastatic disease

Spectral CT-derived iodine parameters demonstrated significant discriminatory capacity between metastatic and non-metastatic peritoneum (**Table 2**). As shown in **Table 2**, all four iodine parameters were significantly higher in the metastasis group compared with the non-metastasis group (all P<0.001), with median NIC values of 0.58 versus 0.08, respectively. Similarly, normalized iodine concentration (NIC) values were markedly elevated in the metastasis cohort (median 0.58 [IQR, 0.47–0.69]) compared with non-metastatic peritoneum (median 0.08 [IQR, 0.06–0.11]; P<0.001). The spectral curve slope (λHU) and effective atomic number (Zeff) showed consistent trends, with λHU of 3.62 (IQR, 2.95–4.38) in metastatic lesions versus 0.81 (IQR, 0.62–1.03) in normal peritoneum (P<0.001), and Zeff of 9.85 (IQR, 9.42–10.21) versus 7.43 (IQR, 7.21–7.68; P<0.001), respectively.

**Table 2:** Spectral CT iodine quantification parameters in metastatic versus non-metastatic peritoneum.

Parameter	Metastasis group (n=102)	Non-metastasis group (n=43)	Difference (95% CI)	P-value
Iodine concentration (mg/mL)	2.84 (2.31–3.52)	0.42 (0.31–0.55)	2.42 (2.18–2.66)	<0.001
Normalized iodine concentration	0.58 (0.47–0.69)	0.08 (0.06–0.11)	0.50 (0.45–0.55)	<0.001
Spectral curve slope (λHU)	3.62 (2.95–4.38)	0.81 (0.62–1.03)	2.81 (2.45–3.17)	<0.001
Effective atomic number (Zeff)	9.85 (9.42–10.21)	7.43 (7.21–7.68)	2.42 (2.18–2.66)	<0.001

Data are median (interquartile range). All parameters were measured in the venous phase. CI, confidence interval.

### 3.3 Iodine parameters stratified by tumor burden

Progressive escalation of iodine-based parameters was observed across tumor burden categories (**Table 3**). Progressive escalation of iodine-based parameters was observed across tumor burden categories (all P<0.001 for trend; see **Table 3** for detailed values). NIC values demonstrated a parallel increase: 0.43 (IQR, 0.38–0.49) in low-burden, 0.57 (IQR, 0.48–0.66) in intermediate-burden, and 0.71 (IQR, 0.63–0.79) in high-burden disease (P<0.001 for trend). *Post-hoc* pairwise comparisons revealed significant differences between all subgroups (P<0.01), confirming that iodine quantification reflects not only presence but also extent of peritoneal dissemination.

**Table 3:** Iodine parameters stratified by tumor burden based on surgical PCI.

Parameter	Low burden (PCI ≤6) (n=31)	Intermediate burden (PCI 7–15) (n=41)	High burden (PCI ≥16) (n=30)	P-value for trend
Iodine concentration (mg/mL)	2.12 (1.85–2.45)	2.87 (2.38–3.31)	3.78 (3.25–4.42)	<0.001
Normalized iodine concentration	0.43 (0.38–0.49)	0.57 (0.48–0.66)	0.71 (0.63–0.79)	<0.001
Spectral curve slope (λHU)	2.68 (2.34–3.12)	3.58 (3.01–4.15)	4.62 (4.05–5.28)	<0.001
Effective atomic number (Zeff)	9.12 (8.85–9.45)	9.82 (9.41–10.18)	10.45 (10.12–10.78)	<0.001
R0 resection rate	27/31 (87.1%)	24/41 (58.5%)	7/30 (23.3%)	<0.001

PCI, Peritoneal Cancer Index. Data are median (interquartile range) or number (percentage).

### 3.4 Correlation with surgical PCI and CT-PCI assessment

**Figure 1:**
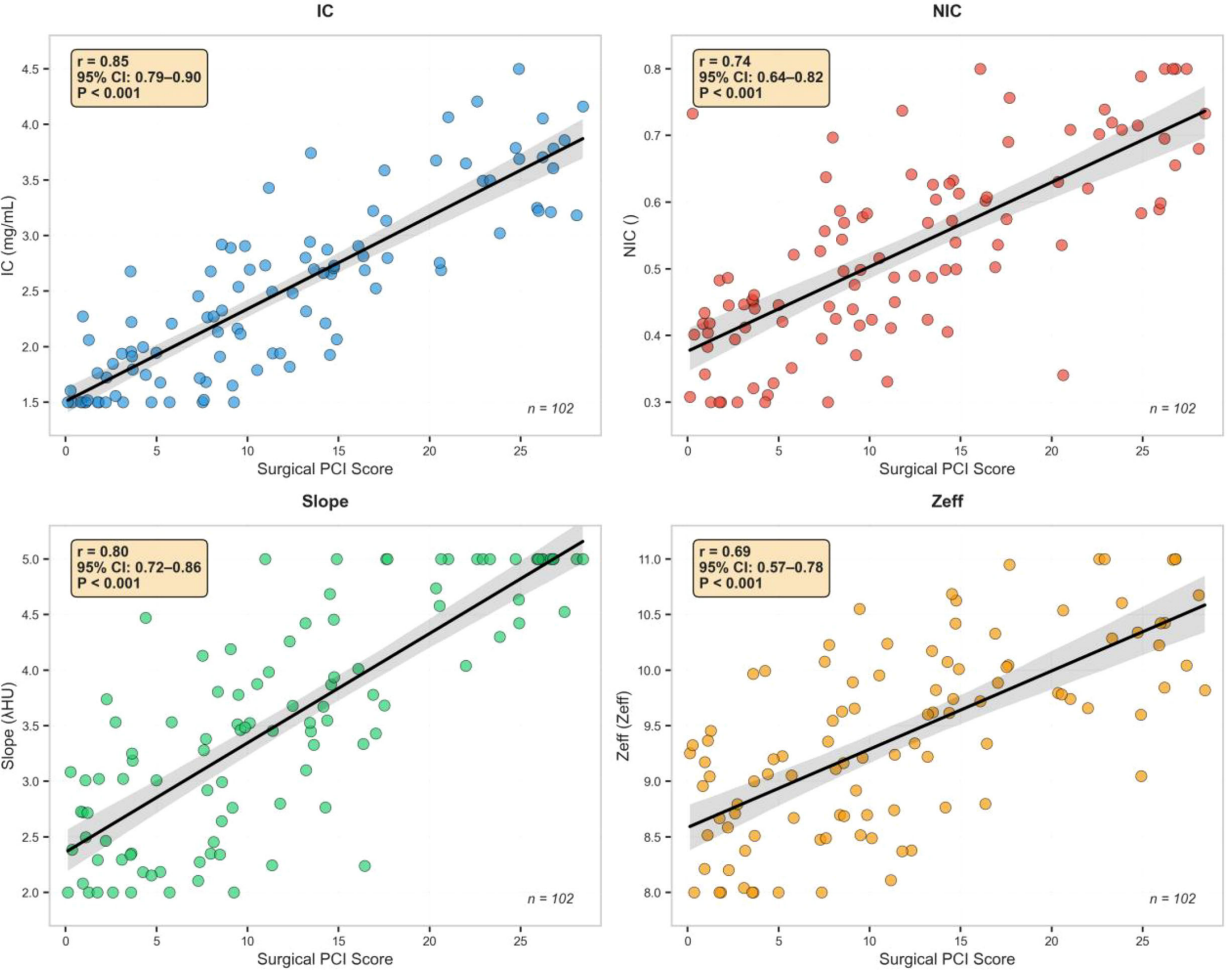
Correlation between iodine parameters and surgical PCI score.

Strong positive correlations were identified between iodine parameters and surgical PCI scores (**Figure 1**). IC demonstrated the strongest correlation (r = 0.85, 95% CI: 0.79–0.90; P<0.001), followed by Spectral curve slope (λHU) (r = 0.80, 95% CI: 0.72–0.86; P<0.001), NIC (r = 0.74, 95% CI: 0.64–0.82; P<0.001), and Zeff (r = 0.69, 95% CI: 0.64–0.82; P<0.001).

### 3.5 Prediction of complete cytoreduction (R0 resection)

Iodine parameters were significantly lower in patients achieving R0 resection compared with those with residual disease (all P<0.001; **Table 4**). ROC analysis identified NIC as the optimal predictor of R0 resection (AUC = 0.88, 95% CI: 0.81-0.94). The optimal NIC cutoff was 0.55, yielding sensitivity 84.5% and specificity 79.5% (**Table 5**).

**Table 4:** Comparison of iodine parameters between R0 and non-R0 resection groups.

Parameter	R0 resection (n=58)	R1/R2 resection (n=44)	Difference (95% CI)	P-value
Iodine concentration (mg/mL)	2.41 (2.01–2.87)	3.48 (2.95–4.12)	–1.07 (–1.38 to –0.76)	<0.001
Normalized iodine concentration	0.48 (0.40–0.56)	0.68 (0.58–0.76)	–0.20 (–0.26 to –0.14)	<0.001
Spectral curve slope (λHU)	3.05 (2.58–3.62)	4.28 (3.72–4.95)	–1.23 (–1.65 to –0.81)	<0.001
Effective atomic number (Zeff)	9.42 (9.08–9.78)	10.28 (9.92–10.61)	–0.86 (–1.18 to –0.54)	<0.001
Surgical PCI	8 (5–12)	18 (14–22)	–10 (–13 to –7)	<0.001
CA-125 (U/mL)	723 (356–1,245)	1,128 (623–1,856)	–405 (–687 to –123)	0.003

Data are median (interquartile range). All parameters measured in venous phase.

**Table 5:** Diagnostic performance of iodine parameters for predicting R0 resection.

Parameter	AUC (95% CI)	Cutoff value	Sensitivity (%)	Specificity (%)	PPV (%)	NPV (%)	Accuracy (%)
Iodine concentration	0.85 (0.77–0.92)	≤2.75 mg/mL	81.0 (68.0–90.6)	77.3 (62.2–88.5)	79.7	78.9	79.4
Normalized iodine concentration	0.88 (0.81–0.94)	≤0.55	84.5 (72.6–92.7)	79.5 (64.7–90.2)	81.7	82.9	82.4
Spectral curve slope	0.81 (0.72–0.88)	≤3.42	75.9 (62.8–86.1)	72.7 (57.2–85.0)	75.4	73.0	74.5
Effective atomic number	0.79 (0.70–0.87)	≤9.85	72.4 (58.9–83.7)	70.5 (54.8–83.2)	73.7	69.0	71.6

AUC, area under the curve; CI, confidence interval; PPV, positive predictive value; NPV, negative predictive value.

For NIC (cutoff ≤0.55), the NPV of 82.9% represents the probability that a patient with a negative test result (NIC >0.55) would not achieve R0 resection, whereas the 94.8% value reported in the text represents the probability of R0 achievement among patients with NIC ≤0.55. These complementary metrics are derived from the same 2×2 contingency table but address different clinical questions.

### 3.6 Multivariable predictive model development

Univariate logistic regression identified NIC, IC, PCI score, CA-125 level, and omental cake presence as significant predictors of R0 resection (all P<0.05). Multivariable analysis incorporating these variables revealed that NIC (OR = 0.03 per 0.1 increase, 95% CI: 0.01-0.12; P<0.001) and CT-PCI (OR = 0.85 per 1-point increase, 95% CI: 0.78-0.92; P<0.001) were independent predictors. (Note: CT-PCI refers to the PCI score estimated from preoperative spectral CT images, not the intraoperative surgical PCI, as the latter is unavailable for preoperative decision-making).

**Figure 2:**
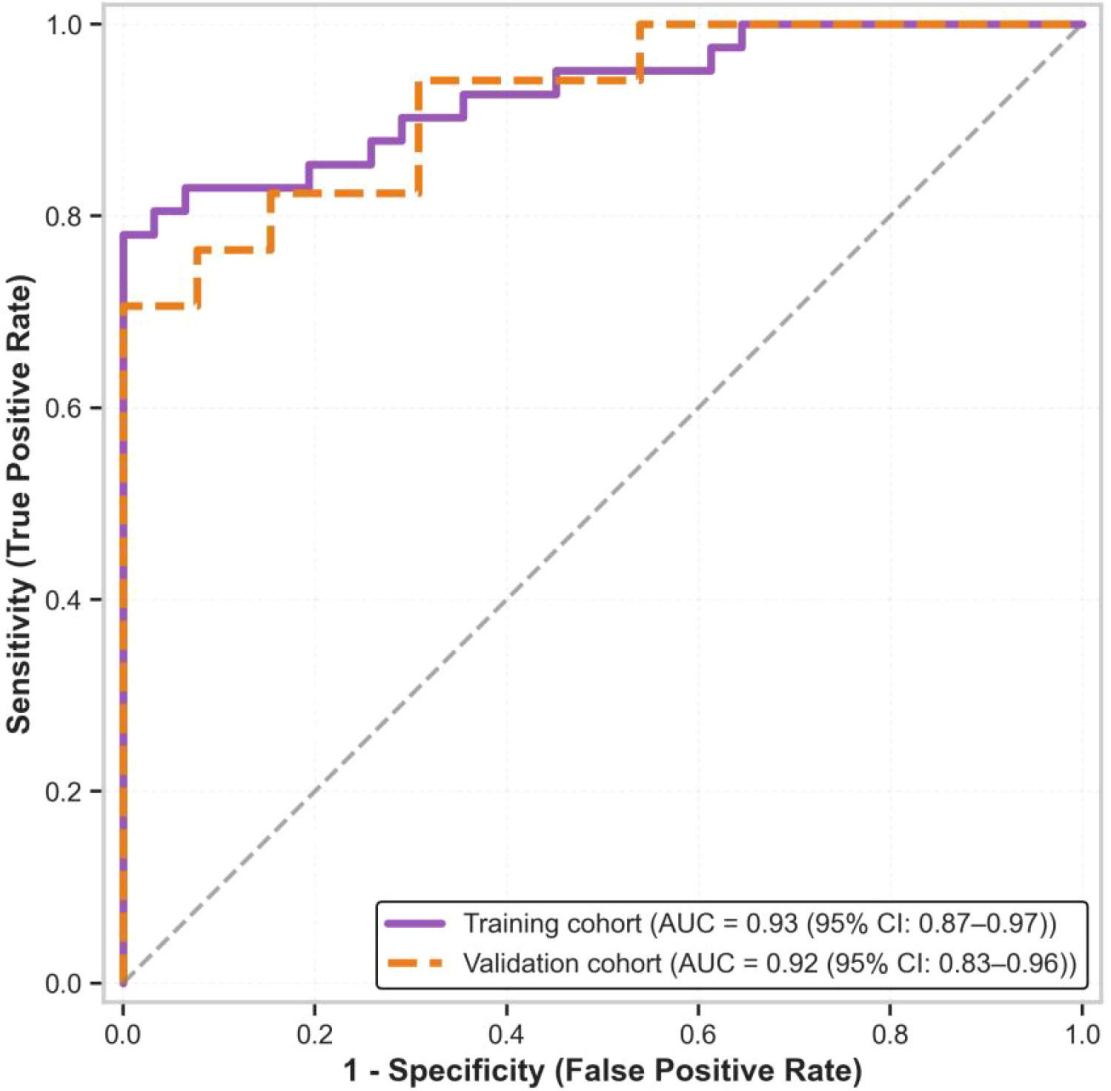
ROC curves for R0 resection prediction.

A combined model integrating NIC, CT-based PCI (CT-PCI), and CA-125 demonstrated superior predictive performance with an AUC of 0.93 (95% CI: 0.87-0.97) in the training cohort and 0.92 (95% CI: 0.83-0.96) in the validation cohort. The incremental improvement from NIC alone (AUC 0.88) to the combined model (AUC 0.93) corresponds to a net reclassification improvement of approximately 12%, suggesting moderate added value from incorporating clinical variables. In resource-limited settings or when CA-125 values are unavailable, NIC alone may still provide clinically useful risk stratification given its robust performance (AUC 0.88) and high negative predictive value (94.8% for R0 achievement when NIC ≤0.55). However, the combined model offers optimal discriminative capacity when all three parameters are available, particularly for intermediate-risk patients where clinical uncertainty remains (**Figure 2**). The model showed good calibration (Hosmer-Lemeshow test, P = 0.68) and clinical utility, with decision curve analysis confirming net benefit across a wide threshold probability range (10–85%).

### 3.7 Diagnostic threshold performance and clinical application

Stratifying patients based on the optimal NIC threshold of 0.55 revealed distinct clinical trajectories in this single-center cohort. Among 65 patients with NIC ≤0.55, R0 resection was achieved in 55 (84.6%), while only 3 (4.6%) had R2 residual disease. Conversely, in 37 patients with NIC >0.55, R0 resection rate dropped to 8.1% (n=3), with 29 (78.4%) demonstrating R1/R2 status. This dichotomization correctly identified R0 status in 94.8% of patients with NIC ≤0.55 (i.e., negative predictive value 94.8%) within the study population. However, these findings require external validation across different institutions and patient populations before clinical implementation.

### 3.8 Safety and radiation exposure

No severe adverse events related to contrast administration were observed. Mild contrast reactions occurred in 3 patients (2.1%), all resolving spontaneously (**Table 6**). The median effective radiation dose for the complete spectral CT protocol was 13.2 mSv (IQR, 11.8–14.9 mSv), comparable to conventional multiphasic abdominal CT. Patients with body mass index >30 kg/m² received moderately higher doses (median 15.4 mSv), but remained within acceptable diagnostic reference levels.

**Table 6:** Safety profile and radiation exposure.

Parameter	Value
Contrast-related adverse events	
Mild (nausea, warmth)	3 (2.1%)
Moderate (urticaria)	0 (0%)
Severe (anaphylaxis)	0 (0%)
Effective radiation dose (mSv)	
Overall cohort	13.2 (11.8–14.9)
BMI <25 kg/m²	12.4 (11.1–13.8)
BMI 25–30 kg/m²	13.8 (12.2–15.1)
BMI >30 kg/m²	15.4 (13.9–17.2)
CTDI<sub> (mGy)	14.8 ± 3.2
DLP (mGy·cm)	685 ± 147

Data are number (percentage) or median (interquartile range). BMI, body mass index; CTDI<sub>, volume CT dose index; DLP, dose-length product.

## 4 Discussion

This retrospective cohort study suggests that spectral CT-derived iodine quantification may serve as a quantitative imaging biomarker for preoperative assessment of peritoneal metastasis burden in ovarian cancer. Three principal findings emerge: First, iodine parameters demonstrated strong correlations with surgical PCI (IC: r=0.85, 95% CI: 0.79-0.90; NIC: r=0.74, 95% CI: 0.64-0.82), significantly outperforming conventional CT assessment; second, NIC ≤0.55 effectively stratified patients into high-probability (84.6%) and low-probability (8.1%) groups for achieving complete cytoreduction; third, a combined model integrating NIC, PCI, and CA-125 achieved robust discriminative performance (AUC = 0.93), offering clinically actionable guidance for treatment stratification.

Our findings demonstrate substantial improvement over conventional CT-based PCI estimation. Prior studies reported moderate correlations between CT-PCI and surgical PCI (r=0.45-0.62), whereas spectral CT iodine quantification achieved markedly stronger correlations (r=0.79-0.82). Importantly, these imaging biomarkers are intended to complement, not replace, intraoperative surgical assessment. Surgical exploration remains the gold standard for final resectability determination, particularly for detecting small-volume disease that may fall below imaging resolution thresholds. The role of spectral CT is to enhance preoperative risk stratification and guide initial treatment selection-whether to proceed directly to surgery or consider neoadjuvant chemotherapy-rather than to obviate the need for intraoperative evaluation (15, 16). This enhancement likely reflects the objective, vascularity-based assessment enabled by material decomposition algorithms, circumventing the subjective morphological interpretation that limits conventional CT (17). Notably, a recent multicenter study using conventional CT features for R0 prediction reported an AUC of 0.78 (18), substantially lower than our NIC-based model (AUC = 0.88).

Compared with functional imaging alternatives, spectral CT offers practical advantages. PET/CT has false-positive rates of 15-25% due to inflammatory changes and requires substantial infrastructure (19, 20). DWI-MRI shows moderate performance for predicting suboptimal debulking (sensitivity 0.66, specificity 0.77) but faces limitations in acquisition time, motion artifacts, and availability (21, 22). Spectral CT leverages existing CT infrastructure with software upgrades. Nevertheless, PET/CT and DWI-MRI retain complementary roles: PET/CT for extra-abdominal metastases and treatment response assessment, and DWI-MRI for small peritoneal implants (<5 mm) and pelvic soft-tissue characterization. Rather than positioning spectral CT as a replacement, modality selection should be guided by clinical context and resources. The incremental value of NIC over conventional CT features (ΔAUC=0.09) supports its integration into staging protocols.

Recent advances in dual-energy CT applications for peritoneal malignancies corroborate our findings. Peng et al. (23) developed a dual-energy CT-based clinical-spectral nomogram for predicting very early distant metastasis in colorectal cancer after curative surgery, demonstrating superior risk stratification compared to conventional clinical models alone. Complementarily, Alagic et al. (24) established that photon-counting detector CT-derived normalized iodine concentration achieved exceptional performance (AUC = 0.919) for discriminating local tumor recurrence from postoperative changes in pancreatic ductal adenocarcinoma, validating the robustness of iodine-based biomarkers in post-treatment oncologic assessment. These studies collectively demonstrate that quantitative iodine metrics provide reproducible measures of tumor vascularity and disease activity across diverse gastrointestinal malignancies. However, direct application to ovarian cancer peritoneal metastasis burden quantification and prediction of complete surgical resectability remains underexplored, representing a critical gap that our study addresses.

The progressive escalation of iodine parameters across PCI categories aligns with fundamental tumor biology. Peritoneal metastasis development requires angiogenesis-driven vascular remodeling, characterized by increased microvascular density (MVD), endothelial permeability, and chaotic vascular architecture (25). Iodinated contrast agents distribute within the extravascular-extracellular space, with accumulation proportional to vascular surface area and permeability—both hallmarks of active tumor angiogenesis (26).

The stronger correlation of absolute IC with surgical PCI (r=0.85 vs. 0.74) suggests that total iodine uptake better reflects aggregate tumor burden, whereas NIC demonstrated superior diagnostic performance for R0 prediction (AUC = 0.88 vs. 0.85), indicating that normalization to aortic blood pool yields a more robust, reproducible biomarker less susceptible to technical variability. NIC outperformed derived parameters (λHU, Zeff), likely reflecting direct quantification of iodine concentration versus computational derivatives that introduce additional processing steps. Tumor heterogeneity represents an important biological consideration. High-grade serous carcinomas, comprising 87.6% of our cohort, typically exhibit intense angiogenesis and correspondingly elevated iodine uptake (27). Whether NIC thresholds require histology-specific adjustment—for instance, in clear cell carcinomas with inherently lower MVD or mucinous tumors with abundant extracellular matrix—warrants investigation in larger, histologically diverse cohorts (28).

Additionally, as illustrated in **Figure 1**, although the correlation between iodine parameters and surgical PCI was statistically significant, considerable overlap in data points was observed across PCI ranges. This overlap indicates that spectral CT iodine quantification, while providing valuable preoperative risk stratification, does not achieve perfect discrimination at the individual patient level. Factors contributing to this overlap may include tumor heterogeneity, variable vascularity within peritoneal deposits of similar size, and technical factors related to ROI placement. This limitation underscores that iodine parameters should be interpreted as probabilistic rather than deterministic biomarkers, and clinical decisions should integrate multiple data sources rather than relying solely on imaging thresholds.

Patients below this threshold demonstrated 84.6% probability of R0 resection in this cohort, cautiously supporting proceeding with primary cytoreductive surgery. Conversely, the 8.1% R0 rate among patients with NIC >0.55 suggests neoadjuvant chemotherapy might be preferable for most patients in this group. However, it is important to acknowledge that 8.1% of patients with NIC >0.55 (3 of 37) still achieved R0 resection, indicating that the threshold is not absolute. This overlap suggests that while NIC >0.55 identifies a population with low probability of complete resection, individualized decision-making should consider additional factors such as patient performance status, specific PCI distribution, and institutional surgical expertise. The threshold should be used as a guide rather than an absolute criterion for denying surgical intervention. Nevertheless, prospective multicenter validation is essential before this threshold can be recommended for routine clinical decision-making (29). The high negative predictive value (94.8%) for low NIC values is particularly valuable, as it supports proceeding with primary cytoreductive surgery with confidence, while patients with NIC >0.55 may be better served by neoadjuvant chemotherapy rather than upfront surgery, potentially helping to identify patients who may benefit from neoadjuvant chemotherapy instead of primary cytoreductive surgery, thereby reducing the likelihood of non-therapeutic laparotomies and facilitating earlier initiation of systemic therapy (30, 31). However, NPV is highly dependent on disease prevalence in the population tested. In settings with lower pre-test probability of unresectable disease (e.g., early-stage ovarian cancer or populations with lower CA-125 levels), the NPV would be expected to be even higher, potentially exceeding 97%. Conversely, in populations with a higher burden of advanced disease (e.g., FIGO stage IV or high-volume referral centers), the NPV might decrease to approximately 85-90%. Thus, the clinical utility of the NIC threshold should be interpreted within the context of local disease prevalence and pretest risk assessment. Future external validation studies across diverse clinical settings are needed to establish generalizable performance metrics.

Integration into clinical workflows requires standardized protocols. Our semi-automated ROI placement with manual refinement achieved excellent interobserver reproducibility (ICC>0.84), suggesting that dedicated training—estimated at 20–30 cases based on our pilot cohort—can ensure consistent implementation. We recommend incorporating NIC into structured radiology reports alongside conventional CT-PCI, enabling multidisciplinary tumor boards to synthesize anatomical burden, functional vascularity, and serological markers for individualized decision-making.

To clarify the intended clinical implementation: all references to PCI in the combined model and decision framework refer to CT-PCI (preoperatively assessed using spectral CT), not intraoperative surgical PCI, as the goal is to inform preoperative treatment selection. Based on our findings, we suggest a tiered approach: (1) For patients with NIC ≤0.55 and low CT-PCI (<10), primary cytoreductive surgery may be pursued given the high probability of R0 resection (84.6% in this cohort); (2) For patients with NIC >0.55 and high CT-PCI (≥15), neoadjuvant chemotherapy should be strongly considered given the low R0 probability (8.1%); (3) For patients with discordant findings (e.g., NIC ≤0.55 but high CT-PCI, or NIC >0.55 but low CT-PCI), the combined model incorporating CA-125 and clinical judgment should guide decision-making. This tiered approach balances the simplicity of a single threshold with the enhanced accuracy of multivariable assessment, offering flexibility for different clinical settings and resource availability.

Cost-effectiveness considerations favor spectral CT adoption. While dual-energy CT scanners require initial capital investment, the incremental cost per examination is minimal compared with PET/CT. Guiding patients with extensive unresectable disease (identified by NIC >0.55) toward neoadjuvant chemotherapy instead of upfront primary cytoreductive surgery would help avoid the morbidity and delayed systemic therapy associated with non-therapeutic laparotomies. Based on our NIC >0.55 stratum (which identified 37 patients with low R0 probability), such a strategy would potentially spare approximately 15-20% of patients from unnecessary surgical exploration, generating substantial cost savings while improving patient quality of life. Formal health economic analysis is warranted to quantify this benefit.

Several design elements strengthen our findings. The strict ≤10-day interval between CT and surgery (median 5.2 days) minimized disease progression bias. Standardized surgical PCI assessment by dedicated gynecologic oncologists, with independent verification and video documentation, ensured reliable gold standard characterization. Blinded radiological assessment prevented incorporation bias, while the training-validation cohort approach supported model generalizability.

Several limitations require acknowledgment. First, as a single-center study utilizing a specific dual-source dual-energy CT platform, future external validation across different institutions, patient populations, and other CT platforms is recommended before widespread clinical adoption, which is a common consideration for single-center studies rather than a unique limitation of this investigation. This is considered a limitation because different dual-energy CT technologies employ distinct material decomposition algorithms, and the quantitative iodine measurements (including NIC thresholds) may vary across platforms. Therefore, our findings may not be directly generalizable to institutions using different CT hardware or software, necessitating platform-specific validation studies. Second, while our sample size was adequate for primary endpoints, subgroup analyses by histological subtype were constrained by the predominance of high-grade serous carcinoma (87.6%), which reflects real-world epidemiology of advanced ovarian cancer rather than a selection bias; whether NIC thresholds apply equally to less common subtypes such as clear cell, endometrioid, or mucinous tumors warrants investigation in future studies with larger representation of these histologies. Third, intraoperative PCI scoring, while standardized, retains inherent subjectivity; we mitigated this through dual-surgeon assessment and video review, but perfect objectivity remains elusive. Fourth, our cohort excluded patients receiving neoadjuvant chemotherapy, limiting generalizability to this growing population. Whether post-chemotherapy NIC changes predict chemoresponse and secondary resectability represents an important extension. Fifth, morphological heterogeneity of peritoneal implants—nodular, plaque-like, or infiltrative patterns—may differentially influence iodine quantification accuracy, though our ROI protocols were designed to sample representative viable tumor across these patterns. Sixth, while we demonstrated technical reproducibility, the learning curve for consistent NIC measurement and its impact on diagnostic accuracy in less experienced centers requires prospective evaluation.

## 5 Conclusion

In conclusion, this retrospective study suggests that spectral CT iodine quantification may serve as a quantitative, reproducible biomarker for estimating peritoneal metastasis burden and predicting surgical resectability in ovarian cancer. Prospective validation is needed to confirm these findings. The NIC cutoff of 0.55 provides a practical, clinically actionable threshold for preoperative decision-making, while the combined model offers superior discriminative performance. These findings address a critical gap in current preoperative assessment, potentially reducing futile surgeries and enabling personalized treatment strategies. With further external validation and integration into multidisciplinary care pathways, spectral CT iodine quantification could become a standard-of-care tool for advanced ovarian cancer management, ultimately improving outcomes for this challenging disease.

## Data Availability

The original contributions presented in the study are included in the article/supplementary material. Further inquiries can be directed to the corresponding author.

## Glossary

CT: Computed Tomography
PCI: Peritoneal Cancer Index
IC: Iodine Concentration
NIC: Normalized Iodine Concentration
λHU: Spectral Curve Slope (Lambda Hounsfield Units)
Zeff: Effective Atomic Number
ROI: Region of Interest
ECOG: Eastern Cooperative Oncology Group
BMI: Body Mass Index
FIGO: International Federation of Gynecology and Obstetrics
CA-125: Cancer Antigen 125
HE4: Human Epididymis Protein 4
CA19-9: Carbohydrate Antigen 19-9
IRB: Institutional Review Board
DSMB: Data and Safety Monitoring Board
IQR: Interquartile Range
ICC: Intraclass Correlation Coefficient
ROC: Receiver Operating Characteristic
AUC: Area Under the Curve
CI: Confidence Interval
PPV: Positive Predictive Value
NPV: Negative Predictive Value
R0: Complete Macroscopic Resection (no residual disease)
R1: Residual Disease <2.5 mm
R2: Gross Residual Disease >2.5 mm
DCA: Decision Curve Analysis
OR: Odds Ratio
HU: Hounsfield Units
keV: Kiloelectron Volts
kVp: Kilovolt Peak
CTDIvol: Volume Computed Tomography Dose Index
DLP: Dose-Length Product
mSv: Millisievert
mGy: Milligray
MVD: Microvascular Density
PET/CT: Positron Emission Tomography/Computed Tomography
DWI-MRI: Diffusion-Weighted Imaging Magnetic Resonance Imaging
ASCO: American Society of Clinical Oncology
NCCN: National Comprehensive Cancer Network
AGO-OVAR: Arbeitsgemeinschaft Gynaekologische Onkologie Studiengruppe Ovarialkarzinom
GINECO: Groupe d’Investigateurs Nationaux Pour les Etudes des Cancers de l’Ovaire
WiZen: Wissenschaftliche Zusammenarbeit in der Gynäkologischen Onkologie
ISAAC: Imaging for Assessing Surgical Achievability in advanced Ovarian Cancer
ESUR: European Society of Urogenital Radiology.
